# Staging Laparoscopy in Gastric Cancer Patients Treated with Curative Intent: A European GASTRODATA Cohort Study

**DOI:** 10.1245/s10434-025-17905-6

**Published:** 2025-07-26

**Authors:** Katarzyna Sędłak, Sebastian Kobiałka, Zuzanna Pelc, Yutaka Endo, Ines Gockel, Johanna van Sandick, Gian Luca Baiocchi, Bas Wijnhoven, Suzanne Gisbertz, Manuel Pera, Paolo Morgagni, Massimo Framarini, Arnulf Hoelscher, Stefan Moenig, Piotr Kołodziejczyk, Guillaume Piessen, Clarisse Eveno, Paulo Matos da Costa, Andrew Davies, William Allum, Uberto Fumagalli Romario, Ricardo Rosati, Daniel Reim, Lucio Lara Santos, Domenico D’ugo, Giovanni De Manzoni, Wojciech Kielan, Paul M. Schneider, Timothy M. Pawlik, Wojciech Polkowski, Karol Rawicz-Pruszyński

**Affiliations:** 1 Department of Surgical Oncology, Medical University of Lublin, Lublin, Poland; 2 Department of Surgery, The Ohio State University Wexner Medical Center and James Comprehensive Cancer Center, Columbus, OH USA; 3 Department of Surgery, Transplant, University of Rochester Medical Center, New York, NY USA; 4 Department of Visceral, Transplant, Thoracic and Vascular Surgery, University Hospital of Leipzig, Leipzig, Germany; 5 Department of Surgical Oncology, The Netherlands Cancer Institute–Antoni Van Leeuwenhoek Hospital, Amsterdam, The Netherlands; 6 Department of Clinical and Experimental Sciences, Surgical Clinic, University of Brescia, and Third Division of General Surgery, Spedali Civili Di Brescia, Brescia, Italy; 7 Department of General Surgery, Erasmus Medical Center, Rotterdam, The Netherlands; 8 Department of Surgery, University Medical Center Amsterdam, Amsterdam, The Netherlands; 9 Cancer Treatment and Quality of Life, Cancer Center Amsterdam, Amsterdam, The Netherlands; 10 Department of Digestive Surgery, Hospital Universitario Del Mar, Barcelona, Spain; 11 Department of General Surgery, Morgagni-Pierantoni Hospital, Forlì, Italy; 12 Contilia Center for Esophageal Diseases, Elisabeth Hospital Essen, Essen, Germany; 13 Geneva University Hospital, Geneva, Switzerland; 14 Department of Surgery, Jagiellonian University Medical College, Kraków, Poland; 15 Department of Digestive and Oncological Surgery, University Lille, and Claude Huriez University Hospital, Lille, France; 16 Faculdade de Medicina da Universidade de Lisboa, Lisbon, Portugal; 17 Department of Upper Gastrointestinal and General Surgery, Guy’s and St Thomas’ Hospital, London, UK; 18 Department of Surgery, Royal Marsden NHS Foundation Trust, London, UK; 19 Digestive Surgery, European Institute of Oncology, IRCCS, Milan, Italy; 20 Department of Gastrointestinal Surgery, IRCCS San Raffaele Hospital Research Institute, Milan, Italy; 21 Department of Surgery, TUM School of Medicine and Health, Technical University of Munich, Munich, Germany; 22 Experimental Pathology and Therapeutics Group, Department of Surgical Oncology, Portuguese Institute of Oncology, Porto, Portugal; 23 Department of General Surgery, Fondazione Policlinico Gemelli, Rome, Italy; 24 General and Upper G.I. Surgery Division, Department of Surgery, University of Verona, Verona, Italy; 25 Department of General and Oncological Surgery, Wroclaw Medical University, Wrocław, Poland; 26 Digestive Oncology Tumor Center and Esophageal Cancer Center, Hirslanden Medical Center Zurich, Zurich, Switzerland

**Keywords:** Gastric cancer, Staging laparoscopy, Multimodal treatment

## Abstract

**Background:**

In current guidelines, staging laparoscopy (SL) is recommended in patients with locally advanced gastric cancer (GC). This study aimed to assess the clinical practice of SL and its association with administration of systemic treatment in a European cohort of GC patients (GASTRODATA).

**Methods:**

In this retrospective cohort study, patients with locally advanced GC who underwent multimodal treatment in 24 European centers were analyzed. Patients with early (cT1) or metastatic GC at diagnosis and those with missing data on chemotherapy administration were excluded.

**Results:**

Of 2558 patients, 1726 were selected, with 562 (32.6%) undergoing SL. Patients who did not undergo SL were older (72 vs. 65 years; *p *< 0.001) and had higher Charlson Comorbidity Index scores (≥ 2: 33.8% vs. 20.5%; *p *< 0.001). These patients had more complications (30.9% vs. 24.4%; *p *= 0.005), higher 90-day mortality (4.7% vs. 2.3%; *p *= 0.017), and were less likely to receive neoadjuvant (35% vs. 78.6%; *p *< 0.001) or adjuvant (27.1% vs. 33.8%; *p *= 0.005) chemotherapy. Non-SL patients had higher rates of serosal invasion (pT4: 38.0% vs. 26.0%; *p *< 0.001) and lymph node metastasis (63.5% vs. 60.4%; *p *= 0.004).

**Conclusions:**

SL was performed in one-third of individuals with locally advanced GC. Absence of SL was associated with higher T-stage discrepancy and decreased utilization of multimodal treatment.

**Supplementary Information:**

The online version contains supplementary material available at 10.1245/s10434-025-17905-6.

Despite a steady decline in the global incidence of gastric cancer (GC), it remains the fifth most commonly diagnosed and the third most lethal malignancy.^1^ It is estimated that up to 40% of newly diagnosed GC cases are diagnosed at the metastatic disease stage.^2^ Among these, over 30% involve peritoneal metastases or free cancer cells in washings collected during staging laparoscopy (SL).^3,4^ According to current European Society for Medical Oncology (ESMO) guidelines, SL is an established part of staging in patients with stage IB–III GC.^5^ Similarly, the National Comprehensive Cancer Network (NCCN) guidelines recommend performing SL with cytological evaluation of the washings in all GC patients with clinical stage T1b or higher.^6^ Due to the low sensitivity of conventional imaging procedures, i.e., computed tomography (CT), positron emission tomography (PET)/CT and magnetic resonance imaging (MRI) in detecting peritoneal metastases, the clinical significance of SL is becoming increasingly recognized.^7–9^ The sensitivity of detecting distant metastases in SL exceeds standard imaging methods and ranges between 64 and 99%.^10^ In addition, in detecting free cancer cells, the advantage of preventing unnecessary laparotomies on unresectable patients has also been proven.^10–13^ Until recently, SL has remained an unstandardized procedure with high heterogeneity in the execution technique and peritoneal fluid assessment in GC patients.^14^ Consequently, several attempts have been made to establish guidelines unifying this procedure.^14,15^

In locally advanced stage (cT2-4N0-3M0), the gold standard for the treatment of patients with GC is multimodal therapy, which includes perioperative chemotherapy (POC) and gastrectomy with D2 lymphadenectomy.^5^ However, based on the GASTRODATA registry, the largest repository of GC patients treated in expert European centers, it was shown that in this population only 44.4% of individuals received neoadjuvant chemotherapy (NAC) and 30.8% received POC.^16^ Despite the introduction of POC into the treatment protocol for locally advanced GC, median overall survival (OS) ranges at around 40% and surgery remains the mainstay of care.^17^ For this reason, the possible benefits of SL in the context of POC compliance may remain undiscovered.

Therefore, the current study aimed to assess the clinical practice of SL and its association with the administration of systemic treatment in a European cohort of GC patients (GASTRODATA). Specifically, we sought to assess the influence of SL and NAC on T-stage discrepancy.

## Methods

### Study Participants, Outcomes, and Definitions

A retrospective cohort study using observational methods was performed, utilizing data from the GASTRODATA registry. All patients undergoing gastrectomy for GC between 2017 and 2022 at the European centers participating in the study were included in the database after obtaining written consent. This dataset was initially established to gather information on complications from specialized centers across 11 European nations from 2017 to 2022.^18^ The research was approved by the Institutional Review Board (KE—0254/331/2018) and the Senior Advisors Board of the Registry, and adhered to the Strengthening the Reporting of Observational Studies in Epidemiology (STROBE)^19^ and Strengthening the Reporting of Cohort Studies in Surgery (STROCCS)^20^ guidelines. All procedures were performed in accordance with the current revisions of the Declaration of Helsinki and were retrospectively registered at ResearchRegistry.com

**Fig. 1:**
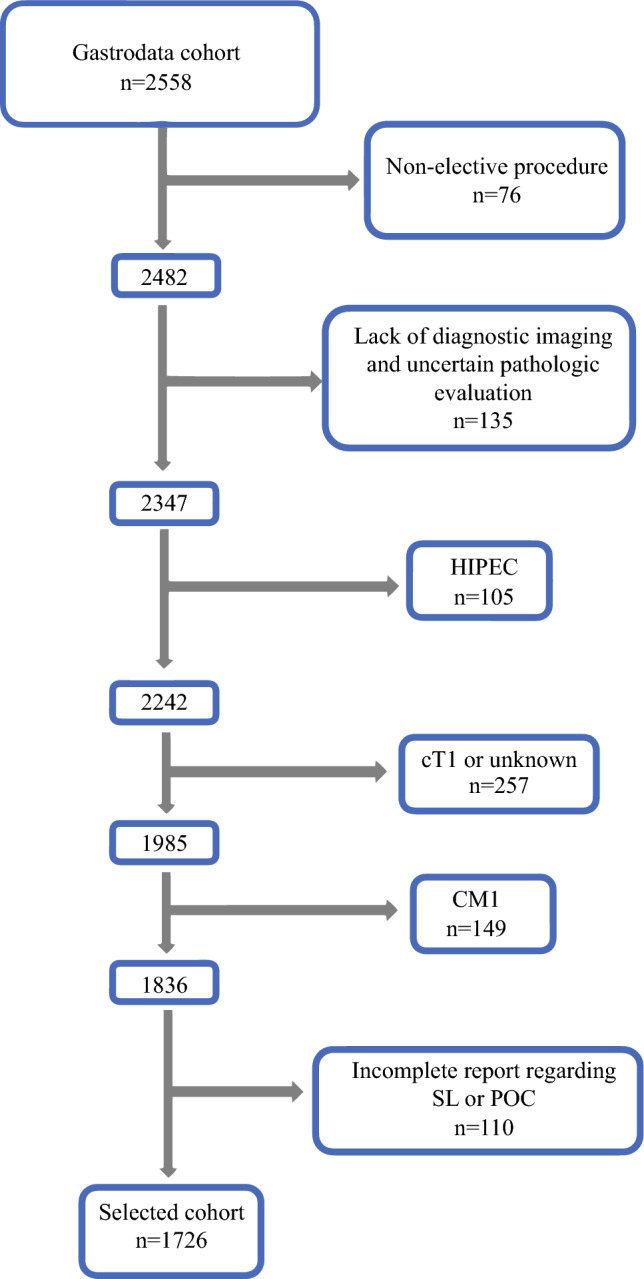
Study selection process. *HIPEC* hyperthermic intraperitoneal chemotherapy, *SL* staging laparoscopy, *POC* perioperative chemotherapy

Patients with stage cT2-4N0-3M0 locally advanced GC, assessed by CT, PET/CT, SL, endoscopic ultrasound, or lavage cytology, based on the 8th edition of the American Joint Cancer on Committee (AJCC) classification system, were included in this study,^21^ as well as patients who underwent curative-intent treatment. The exclusion criteria were non-elective treatment (due to bleeding, perforation, or obstruction), inaccurate clinical staging (lack of diagnostic imaging and uncertain pathologic evaluation), hyperthermic intraperitoneal chemotherapy (HIPEC), incomplete report regarding SL, or preoperative chemotherapy. Patient and disease characteristics were compared between patients who did or did not undergo SL. We additionally evaluated the influence of selected clinical variables on the receipt of NAC, POC, and adjuvant chemotherapy (AC), distinguishing between the entire cohort and SL and non-SL patients (Fig. 1).

The assessment of postoperative complications was carried out by evaluating their occurrence and severity, utilizing the Clavien–Dindo grading system, where severe complications are classified as grade II or higher, or the Comprehensive Complication Index (CCI), where a score above 30 indicates severe complications.^22–24^ The classification of complications followed a structured system from the International Consensus on a Complications List after Gastrectomy for Cancer, detailing 27 types of complications divided into 3 intraoperative, 14 general, and 10 surgical postoperative categories.^18^ An additional measure of study outcome was the 90-day mortality rate, determined by deaths occurring within 90 days post-surgery, irrespective of the patient’s treatment setting. Additionally, T-category discrepancy was assessed and defined as progression from cT stage to (y)pT stage.

### Statistical Analysis

Descriptive statistics were presented as median (interquartile range [IQR]) and frequency (%) for continuous and categorical variables, respectively. Bivariate analyses included the Wilcoxon rank-sum test for continuous variables and Chi-square or Fisher’s exact tests for categorical variables, as appropriate. The trend of NAC for elderly patients with locally advanced GC was assessed using the Mantel–Haenszel trend test. Survival probabilities were estimated using Kaplan–Meier analysis and compared using the log-rank test. To minimize the effect of selection bias of measured covariates on the assessed outcome between the two study groups (SL vs. no SL), propensity score matching (PSM) was performed. A propensity score was calculated for each patient using a logistic regression model, which was fitted for type of treatment using preoperative variables, including age, sex, CCI, American Society of Anesthesiologists (ASA) score, tumor location, and cT stage. Nearest neighbor 1:1 matching without replacement was employed by utilizing a caliper of 0.20. Results relative to covariate subgroups were presented as standardized mean differences (SMDs); SMDs below 0.1 signified minimal disparities between means.^22^ Statistical significance was assessed at α = 0.05, and all statistical analyses were performed using R version 4.2.0 (R Foundation for Statistical Computing, Vienna, Austria).

## Results

### Baseline Characteristics of the Study Group

Overall, 1726 patients selected from the total GASTRODATA cohort of 2558 patients; 562/1726 patients (32.5%) underwent SL. Patients who did not undergo SL were older (72 vs. 65; *p* < 0.001), were more likely to have comorbidities (Charlson Comorbidity Index ≥ 2; 33.8% vs. 20.5%; *p* < 0.001), post- or preoperative complications (30.8% vs. 24.4%; *p* = 0.005), and 90-day mortality (4.7% vs. 2.3%; *p* = 0.017). At the same time, those individuals were less likely to receive NAC (35.1% vs. 78.6%; *p* < 0.001) and AC (27.1% vs. 33.8%; *p* = 0.005) when compared with those undergoing SL. Patients who underwent SL were more likely to receive a PET/CT scan during the diagnostic process (246 [43.8%] vs. 185 [16.1%]; *p* = 0.001) when compared with individuals who did not have SL performed. On pathologic analysis, patients who did not undergo SL were more likely to have serosal invasion (pT4: 38% vs. 26.1%; *p* < 0.001) and lymph node metastasis (pN+: 63.5% vs. 60.5%; *p* = 0.003) when compared with individuals who underwent SL. Not having had an SL was associated with decreased odds of receipt of NAC (odds ratio [OR] 0.14, 95% confidence interval [CI] 0.08–0.24), AC (OR 0.32, 95% CI 0.19–0.53), and POC (OR 0.54, 95% CI 0.31–0.96). The baseline characteristics of the study group are presented in Table 1.

**Table 1:** Baseline characteristics of the study cohort relative to the receipt of staging laparoscopy among patients with locally advanced gastric cancer

	Before PSM				After PSM			
Characteristics	Total [*N* = 1726]	No SL [*n* = 1164]	SL [*n* = 562]	*p*-Value	Total [*N* = 1080]	No SL [*n* = 540]	SL [*n* = 540]	*p*-Value
Age, years [median (IQR)]	70.00 [61.00, 77.00]	72.00 [63.75, 79.00]	65.00 [57.00, 73.00]	< 0.001	66.00 [57.00, 73.25]	66.00 [56.75, 74.00]	66.00 [58.00, 73.00]	0.753
Sex, male	1098 (63.6)	759 (65.2)	339 (60.3)	0.054	658 (60.9)	331 (61.3)	327 (60.6)	0.852
Charlson comorbidity index, 0–1 vs. 2								
0–1	1218 (70.6)	771 (66.2)	447 (79.5)	< 0.001	852 (78.9)	427 (79.1)	425 (78.7)	0.941
≥ 2	508 (29.4)	393 (33.8)	115 (20.5)		228 (21.1)	113 (20.9)	115 (21.3)	
ASA								
1	172 (10.0)	117 (10.1)	55 (9.8)	< 0.001	102 (9.4)	50 (9.3)	52 (9.6)	0.816
2	852 (49.4)	536 (46.0)	316 (56.2)		594 (55.0)	293 (54.3)	301 (55.7)	
3	604 (35.0)	439 (37.7)	165 (29.4)		337 (31.2)	176 (32.6)	161 (29.8)	
4	38 (2.2)	34 (2.9)	4 (0.7)		8 (0.7)	4 (0.7)	4 (0.7)	
5	1 (0.1)	1 (0.1)	0 (0.0)		0 (0.0)	0 (0.0)	0 (0.0)	
Unknown	59 (3.4)	37 (3.2)	22 (3.9)		39 (3.6)	17 (3.1)	22 (4.0)	
Tumor location								
Upper	295 (17.1)	209 (18.0)	86 (15.3)	0.032	165 (15.3)	81 (15.0)	84 (15.6)	0.964
Middle	659 (38.2)	420 (36.1)	239 (42.5)		459 (42.5)	231 (42.8)	228 (42.2)	
Lower	772 (44.7)	535 (46.0)	237 (42.2)		456 (42.2)	228 (42.2)	228 (42.2)	
Diagnostic modality								
CT	1698 (99.2)	1141 (99.2)	557 (99.1)	1	1061 (98.8)	526 (98.5)	535 (99.1)	0.563
EUS	516 (30.1)	326 (28.3)	190 (33.8)	0.024	344 (32.0)	164 (30.7)	180 (33.3)	0.392
PET/CT	431 (25.2)	185 (16.1)	246 (43.8)	< 0.001	333 (31.0)	93 (17.4)	240 (44.4)	< 0.001
cTstatus								
cT2	372 (21.6)	288 (24.7)	84 (14.9)	< 0.001	168 (15.6)	84 (15.6)	84 (15.6)	0.935
cT3	931 (53.9)	594 (51.0)	337 (60.0)		651 (60.3)	328 (60.7)	323 (59.8)	
cT4	423 (24.5)	282 (24.2)	141 (25.1)		261 (24.2)	128 (23.7)	133 (24.6)	
cN status								
cN0	541 (31.3)	391 (33.6)	150 (26.7)	0.015	302 (28.0)	153 (28.3)	149 (27.6)	0.934
cN+	1012 (58.6)	660 (56.7)	352 (62.6)		667 (61.8)	333 (61.7)	334 (61.9)	
cNX	173 (10.0)	113 (9.7)	60 (10.7)		111 (10.3)	54 (10.0)	57 (10.6)	
pT status								
pT1	235 (13.6)	167 (14.3)	68 (12.1)	< 0.001	138 (12.8)	73 (13.5)	65 (12.0)	< 0.001
pT2	262 (15.2)	167 (14.3)	95 (16.9)		160 (14.8)	66 (12.2)	94 (17.4)	
pT3	641 (37.1)	388 (33.3)	253 (45.0)		447 (41.4)	204 (37.8)	243 (45.0)	
pT4	588 (34.1)	442 (38.0)	146 (26.0)		335 (31.0)	197 (36.5)	138 (25.6)	
pN status					0 (0.0)	0 (0.0)	0 (0.0)	
pN0	647 (37.5)	425 (36.5)	222 (39.5)	0.003	397 (36.8)	182 (33.7)	215 (39.8)	
pN1	301 (17.4)	190 (16.3)	111 (19.8)		200 (18.5)	95 (17.6)	105 (19.4)	
pN2	294 (17.0)	191 (16.4)	103 (18.3)		197 (18.2)	95 (17.6)	102 (18.9)	
pN3	484 (28.0)	358 (30.8)	126 (22.4)		286 (26.5)	168 (31.1)	118 (21.9)	
pM status								
pM0	1555 (90.1)	1038 (89.2)	517 (92.0)	0.08	983 (91.0)	485 (89.8)	498 (92.2)	0.202
pM1	171 (9.9)	126 (10.8)	45 (8.0)		97 (9.0)	55 (10.2)	42 (7.8)	
T-stage migration								
Yes	1287 (74.6)	846 (72.7)	441 (78.5)	0.011	821 (76.0)	399 (73.9)	422 (78.1)	0.117
No	439 (25.4)	318 (27.3)	121 (21.5)		259 (24.0)	141 (26.1)	118 (21.9)	
M-stage migration								
Yes	0 (0)	0 (0)	0 (0)	NA	0 (0)	0 (0)	0 (0)	NA
No	1733 (100.0)	1167 (100.0)	566 (100.0)		1080 (100.0)	540 (100.0)	540 (100.0)	
Surgical approach								
Open	1300 (75.3)	895 (76.9)	405 (72.1)	0.034	801 (74.2)	414 (76.7)	387 (71.7)	0.071
Minimally-invasive	426 (24.7)	269 (23.1)	157 (27.9)		279 (25.8)	126 (23.3)	153 (28.3)	
Surgery								
Subtotal gastrectomy	778 (45.1)	533 (45.8)	245 (43.6)	0.419	467 (43.2)	227 (42.0)	240 (44.4)	0.461
Total gastrectomy	948 (54.9)	631 (54.2)	317 (56.4)		613 (56.8)	313 (58.0)	300 (55.6)	
ERAS	887 (51.4)	588 (50.5)	299 (53.2)	0.32	594 (55.0)	305 (56.5)	289 (53.5)	0.359
GCCG compliance	584 (33.8)	422 (36.3)	162 (28.8)	0.003	329 (30.5)	174 (32.2)	155 (28.7)	0.234
Preoperative chemotherapy								
No	876 (50.8)	756 (64.9)	120 (21.4)	< 0.001	407 (37.7)	290 (53.7)	117 (21.7)	< 0.001
Yes	850 (49.2)	408 (35.1)	442 (78.6)		673 (62.3)	250 (46.3)	423 (78.3)	
Preoperative chemotherapy								
ECF	45 (5.3)	22 (5.4)	23 (5.2)	0.047	34 (5.1)	12 (4.8)	22 (5.2)	0.013
FLOT	498 (58.6)	235 (57.6)	263 (59.5)		391 (58.1)	144 (57.6)	247 (58.4)	
FOLFOX	111 (13.1)	54 (13.2)	57 (12.9)		87 (12.9)	30 (12.0)	57 (13.5)	
Other	175 (20.6)	85 (20.8)	90 (20.4)		143 (21.2)	54 (21.6)	89 (21.0)	
TCF	13 (1.5)	11 (2.7)	2 (0.5)		10 (1.5)	9 (3.6)	1 (0.2)	
Unknown	8 (0.9)	1 (0.2)	7 (1.6)		8 (1.2)	1 (0.4)	7 (1.7)	
Postoperative chemotherapy								
No	1221 (70.7)	849 (72.9)	372 (66.2)	0.005	702 (65.0)	342 (63.3)	360 (66.7)	0.278
Yes	505 (29.3)	315 (27.1)	190 (33.8)		378 (35.0)	198 (36.7)	180 (33.3)	
Postoperative chemotherapy								
ECF	23 (4.6)	10 (3.2)	13 (6.8)	0.001	20 (5.3)	7 (3.5)	13 (7.2)	0.006
FLOT	197 (39.0)	105 (33.3)	92 (48.4)		149 (39.4)	65 (32.8)	84 (46.7)	
FOLFOX	125 (24.8)	83 (26.3)	42 (22.1)		93 (24.6)	51 (25.8)	42 (23.3)	
Other	155 (30.7)	113 (35.9)	42 (22.1)		112 (29.6)	72 (36.4)	40 (22.2)	
TCF	5 (1.0)	4 (1.3)	1 (0.5)		4 (1.1)	3 (1.5)	1 (0.6)	
Postoperative radiotherapy	27 (1.6)	21 (1.8)	6 (1.1)	0.504	20 (1.9)	14 (2.6)	6 (1.1)	0.114
Textbook oncological outcome	933 (55.0)	612 (53.4)	321 (58.5)	0.054	625 (58.9)	312 (58.4)	313 (59.4)	0.797
Margin negative resection	1583 (91.7)	1056 (90.7)	527 (93.8)	0.094	1002 (92.8)	495 (91.7)	507 (93.9)	0.18
Lymph nodes harvested ≥ 15	1563 (91.4)	1051 (91.0)	512 (92.1)	0.509	995 (92.9)	503 (93.7)	492 (92.1)	0.391
30-day readmission	54 (3.1)	44 (3.8)	10 (1.8)	0.037	25 (2.3)	15 (2.8)	10 (1.9)	0.418
90-day mortality	68 (3.9)	55 (4.7)	13 (2.3)	0.017	31 (2.9)	19 (3.5)	12 (2.2)	0.274
Prolonged hospital stay	438 (25.9)	297 (26.0)	141 (25.8)	0.953	249 (23.5)	119 (22.3)	130 (24.8)	0.385
Re-intervention at readmission	52 (3.0)	33 (2.8)	19 (3.4)	0.55	30 (2.8)	13 (2.4)	17 (3.1)	0.579
Complication	496 (28.7)	359 (30.8)	137 (24.4)	0.005	275 (25.5)	143 (26.5)	132 (24.4)	0.485
Severe complication	282 (16.3)	198 (17.0)	84 (14.9)	0.298	158 (14.6)	76 (14.1)	82 (15.2)	0.667
CCI [0–100]	0.00 [0.00, 20.91]	0.00 [0.00, 20.91]	0.00 [0.00, 0.00]	0.011	0.00 [0.00, 8.66]	0.00 [0.00, 8.66]	0.00 [0.00, 0.00]	0.652
Delayed gastric emptying	15 (0.9)	14 (1.2)	1 (0.2)	0.029	5 (0.5)	4 (0.7)	1 (0.2)	0.374
Anastomostic leak	127 (7.4)	87 (7.5)	40 (7.1)	0.844	71 (6.6)	31 (5.7)	40 (7.4)	0.326
POPF	38 (2.2)	28 (2.4)	10 (1.8)	0.486	22 (2.0)	12 (2.2)	10 (1.9)	0.83
Bleeding	53 (3.1)	40 (3.4)	13 (2.3)	0.235	28 (2.6)	16 (3.0)	12 (2.2)	0.566
Abdominal complications	60 (3.5)	47 (4.0)	13 (2.3)	0.07	30 (2.8)	17 (3.1)	13 (2.4)	0.579
Pulmonary complications	161 (9.3)	108 (9.3)	53 (9.4)	0.93	90 (8.3)	40 (7.4)	50 (9.3)	0.322
Renal complications	46 (2.7)	31 (2.7)	15 (2.7)	1	30 (2.8)	15 (2.8)	15 (2.8)	1
Cardiac complications	18 (1.0)	13 (1.1)	5 (0.9)	0.803	7 (0.6)	2 (0.4)	5 (0.9)	0.452
Others	90 (5.2)	69 (5.9)	21 (3.7)	0.064	52 (4.8)	33 (6.1)	19 (3.5)	0.064

Data are expressed as *n* (%) unless otherwise specified

*ASA* American Society of Anesthesiologists, *CT* computed tomography, *EUS* endoscopic ultrasound, *PET* positron emission tomography, *ERAS* enhanced recovery after surgery, *CCI* Comprehensive Complication Index, *POPF* postoperative pancreatic fistula, *PSM* propensity score matching, *SL* staging laparoscopy, *IQR* interquartile range, *NA* not available, *GCCG* gastric cancer clinical guidelines defined by national comprehensive cancer network

### Influence of Selected Clinical Variables on Neoadjuvant Chemotherapy Administration

In the group of patients who had SL performed, the administration of NAC was increased by clinical staging of the primary tumor (cT3: OR 2.07, 95% CI 1.20–3.55; and cT4: OR 2.88, 95% CI 1.43–5.89), while in the individuals who did not undergo SL, the administration of NAC was increased both by the clinical stage of the primary tumor and lymph nodes (cT3: OR 1.75, 95% CI 0.99–3.17; cT4: OR 1.92, 95% CI 0.99–3.78; cN1: OR 3.30, 95% CI 2.07–5.36; cN2: OR 3.66, 95% CI 1.84–7.37). However, the administration of NAC was decreased by the patients’ general condition (ASA 3: OR 0.53, 95% CI 0.26–1.07) only in the group without SL (Table 2).

**Table 2:** Influence of selected clinical variables on receipt of neoadjuvant chemotherapy

	GASTRODATA cohort	*p*-Value	Staging laparoscopy	*p*-Value	No staging laparoscopy	*p*-Value
cT						
cT2	Ref		Ref		Ref	
cT3	1.79 (1.25, 2.58)	0.002	2.07 (1.20, 3.55)	0.008	1.75 (0.99, 3.17)	0.058
cT4	2.17 (1.40, 3.37)	0.001	2.88 (1.43, 5.89)	0.003	1.92 (0.99, 3.78)	0.056
cN						
cN0	Ref		Ref		Ref	
cN1	2.08 (1.53, 2.81)	< 0.001	1.62 (0.99, 2.65)	0.055	3.30 (2.07, 5.36)	< 0.001
cN2	1.70 (1.06, 2.74)	0.029	0.70 (0.34, 1.49)	0.353	3.66 (1.84, 7.37)	< 0.001
Surgical approach						
Open	Ref		Ref		Ref	
MIS	1.04 (0.76, 1.41)	0.816	1.01 (0.62, 1.67)	0.973	0.83 (0.52, 1.32)	0.425
CCI	0.99 (0.99, 1.00)	0.053	0.99 (0.98, 1.00)	0.095	0.99 (0.98, 1.00)	0.156
ASA						
1	Ref		Ref		Ref	
2	1.00 (0.62, 1.59)	0.988	1.11 (0.48, 2.35)	0.803	0.89 (0.45, 1.73)	0.725
3	0.63 (0.39, 1.02)	0.061	0.72 (0.31, 1.56)	0.42	0.53 (0.26, 1.07)	0.076
Surgery type						
Subtotal	Ref		Ref		Ref	
Total	1.87 (1.44, 2.45)	< 0.001	1.97 (1.26, 3.11)	0.003	2.20 (1.51, 3.24)	< 0.001
ERAS						
No	Ref		Ref		Ref	
Yes	1.00 (0.77, 1.31)	0.975	1.45 (0.93, 2.26)	0.105	0.93 (0.63, 1.38)	0.736

Data are expressed as OR (95% CI)

*MIS* minimally invasive surgery, *CCI* Comprehensive Complication Index, *ASA* American Society of Anesthesiologists, *ERAS* enhanced recovery after surgery, *Ref* reference, *OR* odds ratio, *CI* confidence interval

### Influence of Selected Clinical Variables on Adjuvant Chemotherapy Administration

In the group of patients who did not undergo SL, the administration of AC was increased by pathological staging of the primary tumor and lymph nodes (cT3: OR 1.16, 95% CI 0.67–2.04; cN1: OR 1.68, 95% CI 1.06–2.69; cN2: OR 1.58, 95% CI 0.79–3.11), in contrast to individuals who underwent SL, whereby the administration of AC was only increased by staging of the primary tumor (pT3: OR 1.74, 95% CI 1.00–3.15). In both groups, AC administration was decreased by postoperative complications (SL group: OR 0.97, 95% CI 0.96–0.98; no SL group; OR 0.98, 95% CI 0.96–0.99; *p* < 0.001). Among patients who did not undergo SL, the administration of AC was also decreased by general condition (ASA 3: OR 0.44, 95% CI 0.22–0.87) and increased by total gastrectomy (OR 1.51, 95% CI 1.03–2.22) (Table 3).

**Table 3:** Influence of selected clinical variables on receipt of adjuvant chemotherapy

	GASTRODATA cohort	*p*-Value	Staging laparoscopy	*p*-Value	No staging laparoscopy	*p*-Value
cT						
cT2	Ref		Ref		Ref	
cT3	1.44 (0.98, 2.15)	0.067	1.74 (1.00, 3.15)	0.057	1.16 (0.67, 2.04)	0.594
cT3	1.25 (0.79, 1.98)	0.348	1.43 (0.74, 2.81)	0.292	1.07 (0.56, 2.07)	0.833
cN						
cN0	Ref		Ref		Ref	
cN1	1.23 (0.90, 1.70)	0.191	0.96 (0.61, 1.50)	0.852	1.68 (1.06, 2.69)	0.03
cN2	1.02 (0.62, 1.66)	0.93	0.67 (0.32, 1.36)	0.279	1.58 (0.79, 3.11)	0.191
Surgical approach						
Open	Ref		Ref		Ref	
MIS	0.90 (0.65, 1.22)	0.496	0.90 (0.58, 1.38)	0.618	0.89 (0.56, 1.41)	0.623
CCI	0.97 (0.96, 0.98)	< 0.001	0.97 (0.95, 0.98)	< 0.001	0.98 (0.96, 0.99)	0.002
ASA						
1	Ref		Ref		Ref	
2	0.78 (0.50, 1.21)	0.266	0.79 (0.43, 1.49)	0.465	0.72 (0.38, 1.36)	0.306
3	0.51 (0.32, 0.82)	0.005	0.57 (0.29, 1.11)	0.097	0.44 (0.22, 0.87)	0.018
Surgery type						
Subtotal	Ref		Ref		Ref	
Total	1.35 (1.03, 1.77)	0.030	1.17 (0.79, 1.73)	0.434	1.51 (1.03, 2.22)	0.035
ERAS	Ref		Ref		Ref	
No	Ref		Ref		Ref	
Yes	0.97 (0.74, 1.27)	0.837	0.97 (0.66, 1.42)	0.860	1.01 (0.68, 1.50)	0.963

Data are expressed as OR (95% CI)

*MIS* minimally invasive surgery, *CCI* Comprehensive Complication Index, *ASA* American Society of Anesthesiologists, *ERAS* enhanced recovery after surgery, *Ref* reference, *OR* odds ratio, *CI* confidence interval

### Influence of Selected Clinical Variables on Receipt of Perioperative Chemotherapy

In the group of individuals who did not undergo SL, the receipt of POC was increased by pathological staging (pT3: OR 1.35, 95% CI 0.72–2.51; pT4: OR 1.92, 95% CI 0.90–4.12; pN1: OR 2.94, 95% CI 1.78–4.88; pN2: OR 2.27, 95% CI 1.08–4.95) and total gastrectomy (OR 2.51, 95% CI 1.62–3.90), but on the other hand, decreased by the occurrence of postoperative complications (OR 0.98, 95% CI 0.97–0.99) and patients’ general condition (ASA 2: OR 0.53, 95% CI 0.20–1.26; ASA 3: OR 0.36, 95% CI 0.13–0.89).

In the group of patients who underwent SL, the receipt of POC was increased by primary tumor pathological stage (pT3: OR 2.06, 95% CI 1.08–3.85; pT4: OR 3.12, 95% CI 1.34–7.53), and total gastrectomy (OR 2.23, 95% CI 1.30–3.90) (Table 4).

**Table 4:** Influence of selected clinical variables on receipt of perioperative chemotherapy

	GASTRODATA	*p*-Value	Staging laparoscopy	*p*-Value	No staging laparoscopy	*p*-Value
cTstage						
cT2	Ref		Ref		Ref	
cT3	1.54 (1.01, 2.34)	0.044	2.06 (1.08, 3.85)	0.026	1.35 (0.72, 2.51)	0.339
cT4	2.25 (1.32, 3.89)	0.003	3.12 (1.34, 7.53)	0.009	1.92 (0.90, 4.12)	0.093
cNstage						
cN0	Ref		Ref		Ref	
cN1	2.00 (1.40, 2.86)	< 0.001	1.45 (0.79, 2.62)	0.224	2.94 (1.78, 4.88)	< 0.001
cN2	1.12 (0.66, 1.95)	0.672	0.47 (0.20, 1.11)	0.079	2.27 (1.08, 4.95)	0.034
Surgical approach						
Open	Ref		Ref		Ref	
MIS	1.03 (0.72, 1.49)	0.875	1.09 (0.60, 2.02)	0.778	0.85 (0.51, 1.42)	0.534
CCI	0.99 (0.98, 1.00)	0.001	0.99 (0.98, 1.00)	0.076	0.98 (0.97, 0.99)	0.005
ASA						
1	Ref		Ref		Ref	
2	0.67 (0.33, 1.25)	0.226	0.94 (0.29, 2.46)	0.9	0.53 (0.20, 1.26)	0.168
3	0.42 (0.21, 0.80)	0.011	0.52 (0.16, 1.37)	0.216	0.36 (0.13, 0.89)	0.034
	2.20 (1.59, 3.05)	< 0.001	2.23 (1.30, 3.90)	0.004	2.51 (1.62, 3.90)	< 0.001
	Ref		Ref		Ref	
	1.12 (0.81, 1.54)	0.499	1.55 ( 0.91, 2.66)	0.107	1.03 (0.66, 1.62)	0.89
	0.78 (0.48, 1.28)	0.323	2.41 (0.79, 8.51)	0.143	0.54 (0.31, 0.96)	0.033
Surgery type						
Subtotal	Ref		Ref		Ref	Ref
Total	2.20 (1.59, 3.05)	< 0.001	2.23 (1.30, 3.90)	0.004	2.51 (1.62, 3.90)	< 0.001
ERAS						
No	Ref		Ref		Ref	Ref
Yes	1.12 (0.81, 1.54)	0.499	1.55 (0.91, 2.66)	0.107	1.03 (0.66, 1.62)	0.890

Data are expressed as OR (95% CI)

*MIS* minimally invasive surgery, *CCI* Comprehensive Complication Index, *ASA* American Society of Anesthesiologists, *ERAS* enhanced recovery after surgery, *Ref* reference, *OR* odds ratio, *CI* confidence interval

### T-Stage Discrepancy Analysis

**Fig. 2:**
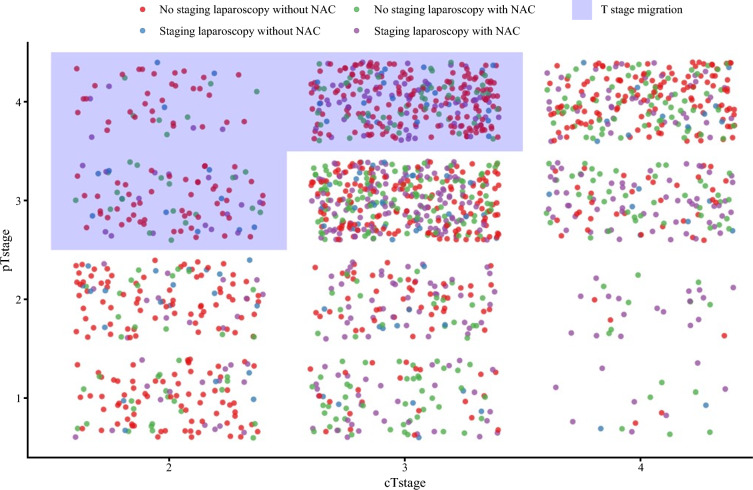
Influence of staging laparoscopy and neoadjuvant chemotherapy administration on T-stage discrepancy. *NAC* neoadjuvant chemotherapy

Overall, 439/1726 (25.4%) patients were reported to have had T-stage discrepancy. Most often, these were individuals who did not undergo SL and did not receive NAC (31.9%), followed by patients who underwent SL and received NAC (29.2%). Individuals who underwent SL and did and did not receive NAC were reported to have T-stage discrepancy in 19.5% and 18.9% of cases, respectively (Table 5, Fig 2).

**Table 5:** Influence of staging laparoscopy and neoadjuvant chemotherapy administration on T-stage discrepancy

		Overall	No NAC without SL	NAC without SL	No NAC with SL	NAC with SL	*p*-Value
*N*		1080	290	117	250	423	
Stage discrepancy	No	821 (76.0)	199 (68.6)	83 (70.9)	200 (80.0)	339 (80.1)	0.001
	Yes	259 (24.0)	91 (31.4)	34 (29.1)	50 (20.0)	84 (19.9)	

Data are expressed as *n* (%)

*NAC* neoadjuvant chemotherapy, *SL* staging laparoscopy

### Comparison of Staging Laparoscopy, Neoadjuvant Chemotherapy, Adjuvant Chemotherapy, and Perioperative Chemotherapy Implementation According to Country

The highest incidence of SL use was reported in the UK, Switzerland, and France, and the lowest incidence was reported in Italy, Poland, and Spain. NAC was most frequently administered in Switzerland, the UK, and Germany, and least frequently in Portugal, Spain, and Italy. POC was registered most frequently in Switzerland, France, and the UK, and least frequently in Portugal, Italy, and Spain (electronic supplementary Figs. 1 and 2)

## Discussion

Despite the presence of SL in global guidelines and its indisputable role in clinical staging, its use is still not systematized and its role sufficiently proven.^5,6,21^ The effect of these inconsistencies can be seen in the constantly emerging national studies that attempt to elucidate the cause of SL omission.^12,15,25–27^ A parallel conclusion applies to the use of NAC and POC instead of postoperative treatment alone. Although the benefits in terms of OS and disease-free survival were reported more than 2 decades ago, multimodal treatment in this form is still not commonly adopted, as shown in a recent GASTRODATA ancillary study on textbook oncological outcome, which indicated that in only 44.4% of patients was stage-adherent guideline compliance fulfilled.^16^

The current study shows that despite collecting data from expert and experienced centers in Europe, where most GC cases are diagnosed at an advanced stage, only 32.7% of patients underwent SL. Recent data from a Dutch analysis corroborated the upward tendency in the utilization of SL, documenting an escalation from 19.6% in 2016 to 32.3% by 2021. Despite this progressive increase, the prevalence of SL implementation within The Netherlands remains suboptimal and below expected clinical standards,^15^ but, at the same time, was expanded during the same period (from 25 to 31%). Furthermore, 37.6% of patients were found to be unresectable during SL, which prevented unnecessary laparotomies in the future and made it possible to adjust appropriate multimodal treatment.^15^ Unfortunately, due to the pertinence and purpose of constructing the GASTRODATA registry, we do not have information on SL that subsequently disqualified patients from surgical treatment.

This study showed that SL was more likely to be omitted in older patients, those with more comorbidities, and those who did not undergo PET/CT during the diagnostic process. Consequently, these patients were less likely to receive NAC and were more likely to have lymph node and distant metastases. In comparison, a Japanese study evaluated elderly GC patients who were and were not treated with NAC. The findings indicated that those receiving NAC had a higher likelihood of better OS rates (75% vs. 36%; *p* = 0.015), demonstrating the safety and efficacy of multimodal therapy in this group.^28^ However, a Dutch study evaluating patients over 75 years of age proved that in this group, patients who received NAC were less likely to undergo gastrectomy.^29^ This demonstrates the complexity of the challenge of applying NAC and SL to elderly patients with GC.

Patients who did not undergo SL exhibited a greater frequency of postoperative complications and an elevated 90-day mortality rate. This may be attributed to imprecise clinical staging, as evidenced by T-stage discrepancy analysis. The reason for this could also be due to worse general condition, older age, or comorbidities. Nonetheless, the guidelines do not exempt SL among such patients. Specifically, those who did not receive SL were more likely to experience inaccurate clinical assessment and may have consequently received suboptimal treatment due to ineligibility for multimodal treatment at the preoperative level. Similarly, in a study based on the National Cancer Database (NCDB), Ju et al. reported that of 4224 stage cT1-T2 GC patients, 1675 (39.7%) were understaged.^30^ Occult nodal positivity (cN0→pN1 or N2) was the most common reason for understaging (73.7%), while larger tumor size (cT1/2→pT3/4) accounted for 20.6% of staging errors, and occult metastatic disease (cM0→pM+) accounted for 5.7%. The authors reported that factors associated with an increased likelihood of inappropriate staging were cT2 tumors, overlapping tumor sites, and non-well-differentiated tumor grades. Similarly, a multicenter Polish study showed that among the Eastern European population, serosal involvement (cT4) and diffuse histological type were independent predictors of peritoneal metastases, which could be discovered during SL.^31^

Given the results of this study and the Textbook Oncological Outcome in European GASTRODATA study,^16^ it is suspected that the low rate of patients undergoing SL may be related to the low overall rate of NAC and POC administration in the European population.

Future prospective studies are warranted to explore tailored strategies for implementing SL and multimodal therapy in elderly or comorbid patients, who may be at increased risk for overtreatment or treatment omission. These studies should aim to define clinical, biological, and functional thresholds that better inform patient selection and optimize the risk-benefit balance. Incorporating geriatric assessments, quality-of-life metrics, and molecular profiling could further refine decision making in this complex subgroup. Our findings, which demonstrate significant differences in treatment pathways and outcomes among older and comorbid patients, underscore the need to prioritize this population in future research and policy discussions.

This study has several limitations that should be considered. First, the retrospective design of the study limited the ability to assess the potential reasons for SL omission, as it was only possible to exclude patients who were operated on non-electively due to life-threatening conditions. Moreover, as the GASTRODATA registry was initially created to mainly monitor complications after GC surgery,^18,32^ precise clinical, pathological staging information and multimodal treatment assessment were limited. The registry does not contain information on patients classified as palliative, and the location of distant metastases at staging or how they were detected. Nonetheless, despite the study’s limitations, the findings, which are also consistent with current reports from the West, appear to offer important insight.

## Conclusion

In the current study that included European GASTRODATA registry patients, SL was performed in only one-third of individuals with locally advanced GC. Absence of SL was associated with a higher rate of T-stage discrepancy and decreased utilization of multimodal treatment. These data warrant an in-depth study on the causes of low adherence to current guidelines.

## Supplementary Information

Below is the link to the electronic supplementary material.

Supplementary file1 (DOCX 532 KB)
