# Supplementary material for: Staging Laparoscopy in Gastric Cancer Patients Treated with Curative Intent: A European GASTRODATA Cohort Study
Source: Ann Surg Oncol. 2025 Jul 26;32(10):7615–26. doi: 10.1245/s10434-025-17905-6 (PMC12454547; doi:10.1245/s10434-025-17905-6)

**Supplementary Figure 1.** Comparison of staging laparoscopy, neoadjuvant chemotherapy, adjuvant chemotherapy and perioperative chemotherapy implementation for particular countries.

**
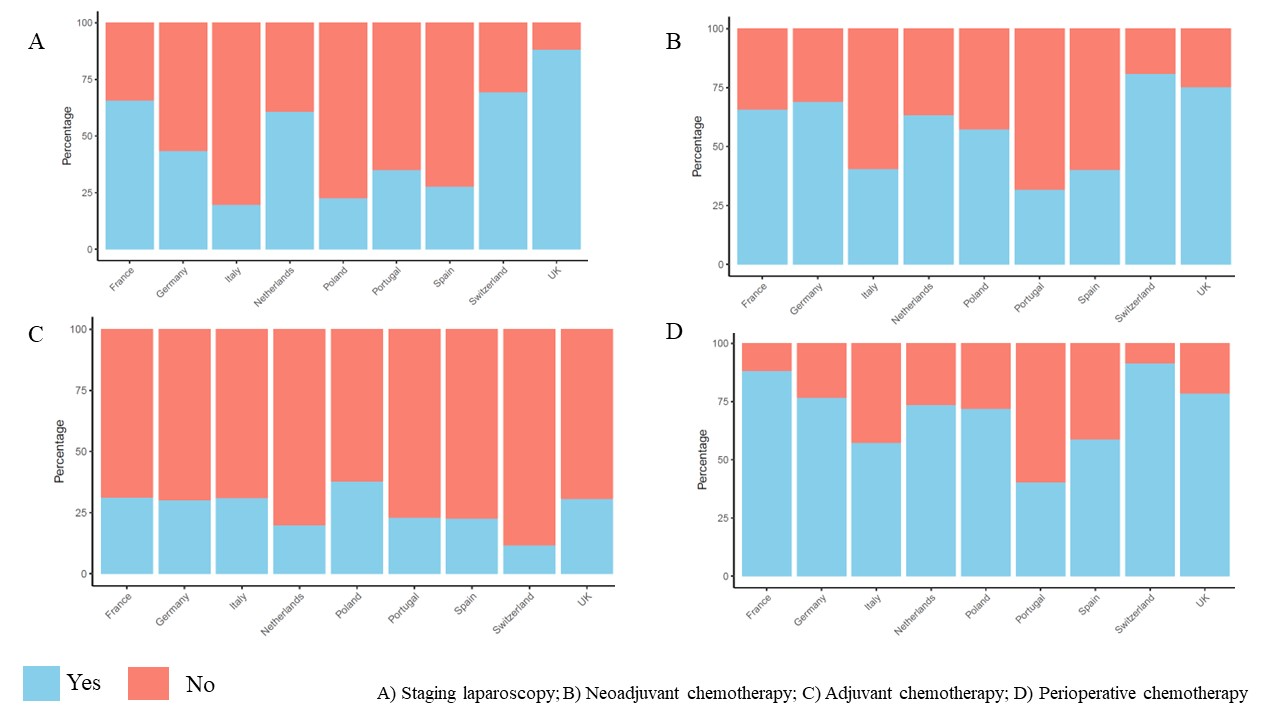
**

**Supplementary Figure 2.** Map of European Participant Countries comparing staging laparoscopy and chemotherapy administration.


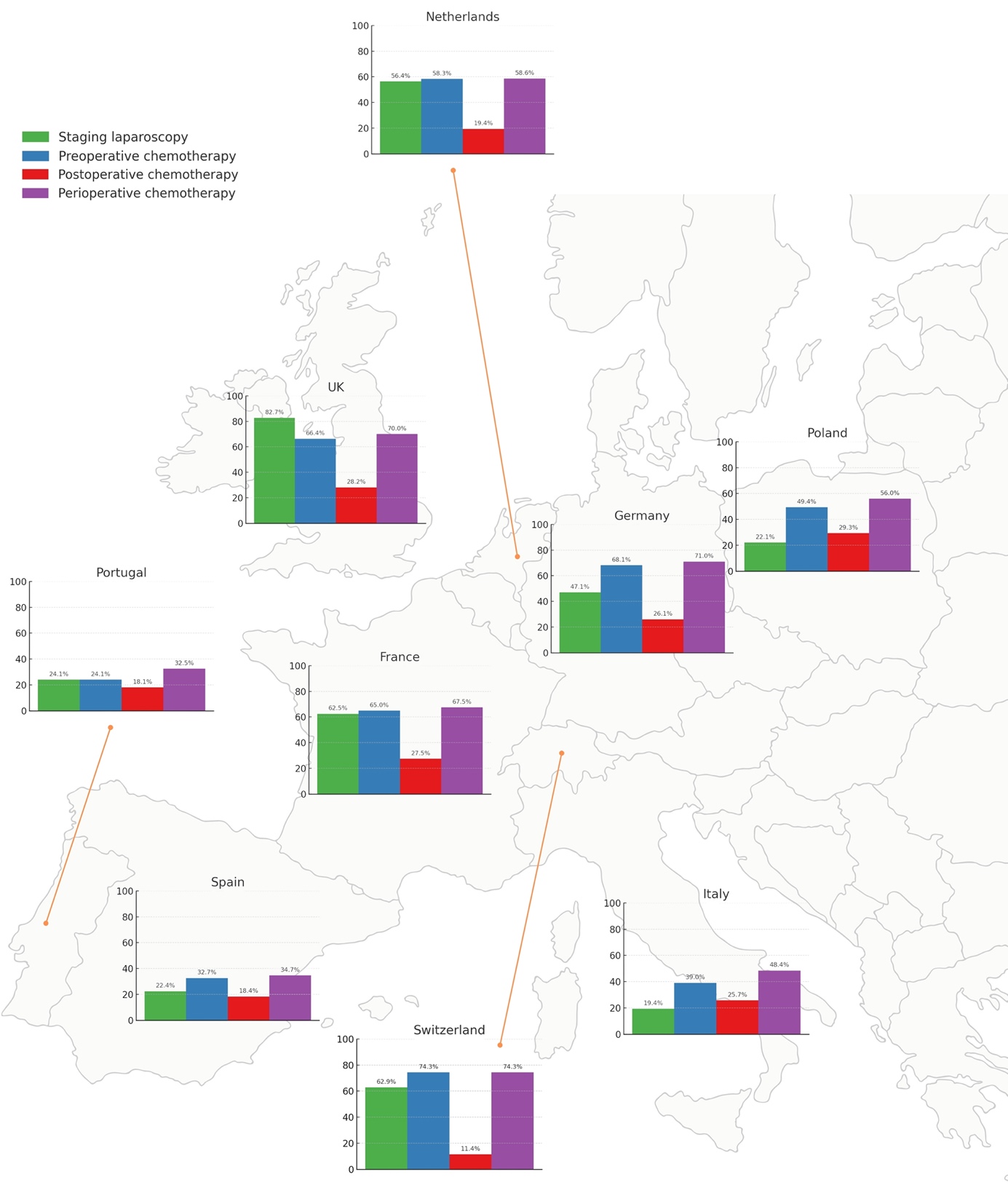

Supplement: Supplementary file 1 — Supplementary file1 (DOCX 532 KB) [file 10434_2025_17905_MOESM1_ESM.docx]
